# Stance leg and surface stability modulate cortical activity during human single leg stance

**DOI:** 10.1007/s00221-021-06035-6

**Published:** 2021-02-11

**Authors:** Daniel Büchel, Tim Lehmann, Sarah Ullrich, John Cockcroft, Quinette Louw, Jochen Baumeister

**Affiliations:** 1 Exercise Science and Neuroscience Unit, Department of Exercise and Health, Faculty of Science, Paderborn University, Warburger Straße 100, 33098 Paderborn, Germany; 2 Department of Child and Adolescent Psychiatry and Psychotherapy, TU Dresden, Dresden, Germany; 3 Neuromechanics Unit, Stellenbosch University, Cape Town, South Africa; 4 Division of Physiotherapy, Department of Health and Rehabilitation Sciences, Stellenbosch University, Cape Town, South Africa

**Keywords:** Postural control, Electroencephalography, Mobile brain imaging, Independent Component Analysis

## Abstract

Mobile Electroencephalography (EEG) provides insights into cortical contributions to postural control. Although changes in theta (4–8 Hz) and alpha frequency power (8–12 Hz) were shown to reflect attentional and sensorimotor processing during balance tasks, information about the effect of stance leg on cortical processing related to postural control is lacking. Therefore, the aim was to examine patterns of cortical activity during single-leg stance with varying surface stability. EEG and force plate data from 21 healthy males (22.43 ± 2.23 years) was recorded during unipedal stance (left/right) on a stable and unstable surface. Using source-space analysis, power spectral density was analyzed in the theta, alpha-1 (8–10 Hz) and alpha-2 (10–12 Hz) frequency bands. Repeated measures ANOVA with the factors leg and surface stability revealed significant interaction effects in the left (*p* = 0.045, *η*_*p*_^2^ = 0.13) and right motor clusters (*F* = 16.156; *p* = 0.001, *η*_*p*_^2^ = 0.41). Furthermore, significant main effects for surface stability were observed for the fronto-central cluster (theta), left and right motor (alpha-1), as well as for the right parieto-occipital cluster (alpha-1/alpha-2). Leg dependent changes in alpha-2 power may indicate lateralized patterns of cortical processing in motor areas during single-leg stance. Future studies may therefore consider lateralized patterns of cortical activity for the interpretation of postural deficiencies in unilateral lower limb injuries.

## Introduction

Postural control represents the ability to monitor and adjust the position and alignment of the body in space and is essential for almost all motor activities of daily life, by representing the ability of monitoring body position and alignment in space. The components of postural orientation and postural stability involve multimodal interactions of the musculoskeletal and neural systems (Shumway-Cook and Woollacott 2012). Postural orientation incorporates visually- and vestibular-guided processes which monitor the interrelationship between body segments relative to the environment. In contrast, postural stability processes predominantly incorporate somatosensory information to control the center of mass in relationship to the base of support (Shumway-Cook and Woollacott 2012). The extent to which the brain plays a role in these processes remains unclear and is an active area of research.

Various neuroimaging studies have already demonstrated that the brain may actively contribute to postural stability in response to changing sensorimotor demands (Mierau et al. 2017; Solis-Escalante et al. 2019; Varghese et al. 2019; Gebel et al., 2020). In conditions with modulations to the base of support, previous findings have revealed increased cortical activity in frontal (Tse et al. 2013; Hülsdünker et al. 2015), motor (Varghese et al., 2015), parietal (Hülsdünker et al., 2016a) and occipital cortical areas (Ouchi et al. 1999; Slobounov et al. 2009). Thus, increased excitability in these cortical areas may reflect enhanced cortical alertness for compensatory postural adjustments in response to naturally occurring challenges of static postural stability (De Waele et al. 2001; Slobounov et al. 2009; Solis-Escalante et al. 2019; Varghese et al. 2019; Lehmann et al. 2020).

Mobile electroencephalography (EEG) has frequently been used to investigate cortical processing related to postural control, because the high mobility and enhanced portability allows investigations of cortical activity during upright stance (Wittenberg et al. 2017). Most of these studies have typically focused on the quantification of immediate brain dynamics in response to mechanical perturbation of postural stability or sustained regulation of cortical activity during challenging continuous balance tasks (Wittenberg et al. 2017), but investigations towards potential hemispheric activation patterns are lacking.

A better understanding of sensorimotor patterns and how these are altered amongst persons with unilateral lower-limb injuries is particularly important for developing individualized approaches to neuromuscular rehabilitation. Previous studies have shown that lateralized motor tasks evoke desynchronized activity in the contralateral sensorimotor areas, as well as a synchronized activity in ipsilateral sensorimotor areas after movement onset (Ramos-Murguialday and Birbaumer 2015). It was assumed that focal desynchronization of task-relevant brain regions, accompanied by a synchronization of non-relevant contra- and ipsilateral brain regions, may increase the gain of the functionally required regions for the specific motor task (Alegre et al. 2004). Albeit unilateral motor tasks performed with the feet evoke less lateralized cortical activation compared to upper limb tasks, a tendency of contralateral cortical representation was reported in fMRI studies (Kapreli et al. 2006). Edwards and colleagues (Edwards et al. 2018) found that alpha desynchronization in the EEG power spectrum was more prominent on the right hemisphere during single-leg stance on the right leg, which supported a hemispheric asymmetry during continuous single-leg postural tasks. In the framework of lower-limb injuries, interaction patterns of stance leg on cortical contributions might help to better understand disturbed unilateral postural control. However, no studies have systematically analyzed leg-dependent modulations of cortical activation during single-leg stance yet.

When investigating cortical contributions to postural control, frequency oscillations in the theta (4–7 Hz) and alpha (8–12 Hz) range have already been attributed a functional role during single-leg continuous balance tasks with changing surface stability (Hülsdünker et al. 2015, 2016b). Theta oscillations are typically associated with working memory and cognitive processes in the frontal cortex of the brain (Klimesch 1999; Sauseng et al. 2010). In the context of postural control, higher frontal theta power has been reported along with enhanced postural sway during tandem stance, single leg stance or stance on an unstable base of support (Slobounov et al. 2009; Sipp et al. 2013; Varghese et al. 2014; Hülsdünker et al. 2015). As also suggested by Hülsdünker et al. (2016a, b), elevations of frontal theta power during postural tasks may function as a marker of focused attention and error detection when postural equilibrium is challenged. Whereas theta is predominantly reported in conjunction with cognitive processes in the fronto-midline of the brain, alpha oscillations are linked to task-specific information processing and cortical alertness. Alpha oscillations are inversely related to the activation of neuronal populations in sensorimotor areas of the brain (Pfurtscheller and Lopes 1999). Functionally separated into alpha-1 (8–10 Hz) and alpha-2 (10–12 Hz) sub-frequencies, alpha power seems to decrease with higher demands on postural control (Del Percio et al. 2007; Babiloni et al. 2014). While alpha-1 has been suggested to reflect increased thalamo-cortical information transfer and global cortical activation, alpha-2 power was associated with movement execution and task-related information processing based on cortico-cortical interactions (Sauseng et al. 2005; Klimesch et al. 2007). Therefore, an observation of lateralized decreases of alpha-2 power in sensorimotor areas during single-leg stance may provide further insights into cortical processes related to postural control.

Therefore, the present study aimed to examine leg-dependent hemispherical cortical activation during single-leg stance in healthy young adults. For a methodological validation of leg-dependent hemispherical activation, participants performed single-leg stances on either leg. Furthermore, participants were asked to perform single-leg stance on both stable and unstable surfaces to investigate whether task-specific demands are subserved by lateralized cortical activation patterns. In this regard, the present study will provide a deeper insight into cortical activation related the maintenance of postural equilibrium during single-leg stance.

## Methods

### Ethics

The study was approved by the Ethics Committee of the Stellenbosch University (N16/05/068) and was designed according to the Declaration of Helsinki. Written informed consent was obtained from all participants. Participants were remunerated for their expenditure of time.

### Participants

In total, 22 healthy participants were initially recruited in this experiment. One participant reported muscle soreness in the middle of the experiment and was excluded from further analysis. Finally, the remaining 21 participants (22.43 ± 2.23 years; 77.31 ± 8.63 kg; 178.9 ± 7.71 cm) completed a health questionnaire before the testing and the kicking leg (right: 100%) was determined by a revised Waterloo Footedness Questionnaire-Revised (van Melick et al. 2017). All participants reported their right leg as their kicking leg. To better understand spatial organization by means of ipsi- and contralateral hemispheric activation patterns within the cortex, kicking and standing leg will further be referred to as the right leg and left leg. Volunteers who had suffered from severe lower limb injuries in the past suffered musculoskeletal pain in the lower quadrant or other neurological conditions, which contribute to postural imbalances, as well as volunteers who had performed lower body training during the last 24 h before the study were not eligible for participation.

### Procedures

Participants were asked to step on the force platform (FP) to perform four blocks of single-leg stance. Participants started on the left (SSL, 50%) or right leg (SSR, 50%) on a stable, then an unstable surface on the left (SUL) or right leg (SUR), followed by single-leg stance on the other leg on a stable (SSL, respectively, SSR) and unstable surface (SUL, respectively, SUR). Each block consisted of 5 repetitions, each with a duration of 30 s. The participants randomly started the experiment on the right leg or left leg to avoid order effects. The position of the non-stance leg during all SLS was instructed to be held in approximately 90° of knee flexion. Subjects were instructed to avoid compensatory arm movement by holding the arms close to the body. In case of excessive arm movement, the investigator gave feedback and repeated the instructions in between trials and affected trials were excluded from the analysis. Participants were given breaks of 30 s after each repetition and one minute after each block. Participants were asked to stand still and in a relaxed posture to prevent muscle artifacts in the recorded scalp data.

### Postural performance and analysis

Center of pressure data for postural performance was captured using a force platform (FP6090-15, Bertec Corporation, Columbus, USA) at a sampling rate of 1000 Hz using the Vicon Nexus software (Version 2.8.2, Vicon Motion Systems, Oxford, UK). During SUR and SUL participants stood on a foam Pad (Balance Pad Elite, Airex AG, Switzerland) to create an unstable surface. A foam pad (*⍴* = 0.0558 g/cm^3^; *V* = 50 × 41 × 6 cm; m = 700 g) was placed on top of the force platform, which was calibrated after each block with the foam pad on its surface to remove any force offsets from the pad or electromechanical drift. Data was stored offline for further analysis, which was done in MATLAB (Version 2019b, Mathworks Inc., Natick, USA). Repetitions with falls as well as participants with less than two valid trials per condition were excluded from the study (*n* = 5). A fall was defined as either touching the floor with the raised foot or moving the weight-bearing foot to maintain balance. Afterward, data were resampled at 100 Hz, and a polyphase filter and anti-aliasing (lowpass) FIR filter was implemented (Duarte et al. 2011). To exclude the initiation and stopping phase, the first three and last three seconds of each trial were removed. Sway velocity (SV, average speed of COP along its path in cm/s) and area of sway (AOS, 95% confidence ellipse in cm^2^) of all trials per condition for each participant was calculated using a MATLAB custom script.

### EEG recordings and analysis

64 active EEG electrodes and tight-fitting caps (Brain Products GmbH, Gilching, Germany), according to the individually measured head circumferences, were used to measure brain activity. Due to transmitters fixed to a small bag back, which the participants were wearing during the investigation, signals were sent to a wireless amplifier (MOVE, Brain Products GmbH, Gilching, Germany) preventing artifacts moving cables and increase participants degrees of freedom. The EEG montage was used in accordance with the international 10–20 system and online referenced to FCz. To synchronize the posturography with EEG, a triggering pulse was delivered to the EEG amplifier at the start and end of each trial during postural data collection. Electrode impedance was maintained below 5 kΩ with a sampling rate of 1000 Hz, and data were stored for offline analysis.

EEG recordings were analyzed with the EEGLAB toolbox 14.1 running in MATLAB 2019b (Mathworks Inc., Natick, MA, USA). Relevant data was run through a processing pipeline that has already been applied in previous investigations (Anders et al. 2018; Lehmann et al. 2020), starting with a Cleanline plugin to remove sinusoidal noise (Mullen 2012). Afterward an FIR band-pass filter from 3 to 30 Hz was applied and data were re-referenced to the common average as well as down-sampled to 256 Hz from 1000 Hz. After pre-processing, artifacts were manually rejected by one experienced observer. After removing non-stereotype artifacts, adaptive mixture independent component analysis (ICA) algorithm (Palmer et al. 2011) was run to decompose data into maximally independent components (ICs) and to differentiate brain from non-brain electrical activity (Onton and Makeig 2006). DIPFIT plugin (Oostenveld and Oostendorp 2002) was used to approximate spatial source for all IC in a standard MRI head model. Only IC with dipoles inside the head model and residual variance (RV) of less than < 15.00% were considered for the analysis (Onton and Makeig 2006). The rejection of non-cortical components was based on the visual inspection of scalp maps, power spectra, dipole location, and time-domain activity of each IC.

IC labeled as brain components were precomputed and pre-clustered with a k-means algorithm based on IC scalp map, power spectrum and dipole location. Only clusters that included IC from more than half of the participants were further considered. Dipoles greater than three standard deviations (SD) from the mean dipole of the six final clusters were assigned to an outlier cluster. Continuous preprocessed EEG data was then split into the four conditions (SSR, SUR, SSL, SUL) and averaged over all data points per condition to compute mean frequency power of theta (4–7 Hz), alpha-1 (8–10 Hz) and alpha-2 (10–12 Hz) band for each condition and each cluster.

### Statistics

Statistical analysis was performed using SPSS Statistics 24.0 (IBM Corp., USA). The significance level was set at *p* < 0.05. A two-way repeated-measures ANOVA design with two independent variables was chosen to calculate main- and interaction effects of stability (stable, unstable) and leg (right/left). The test was applied on SV and AOS. ANOVA on the factors stability and leg was also run on theta in the prefrontal and fronto-central cluster and on alpha-1 and alpha-2 frequency bands in the prefrontal, fronto-central, left-motor, right-motor, left-parietal-occipital, and right-parietal-occipital clusters. For analysis of cortical activity, only mean values of valid trials were included. Normal distribution was confirmed using Shapiro–Wilk tests.

## Results

### Postural performance

**Fig. 1:**
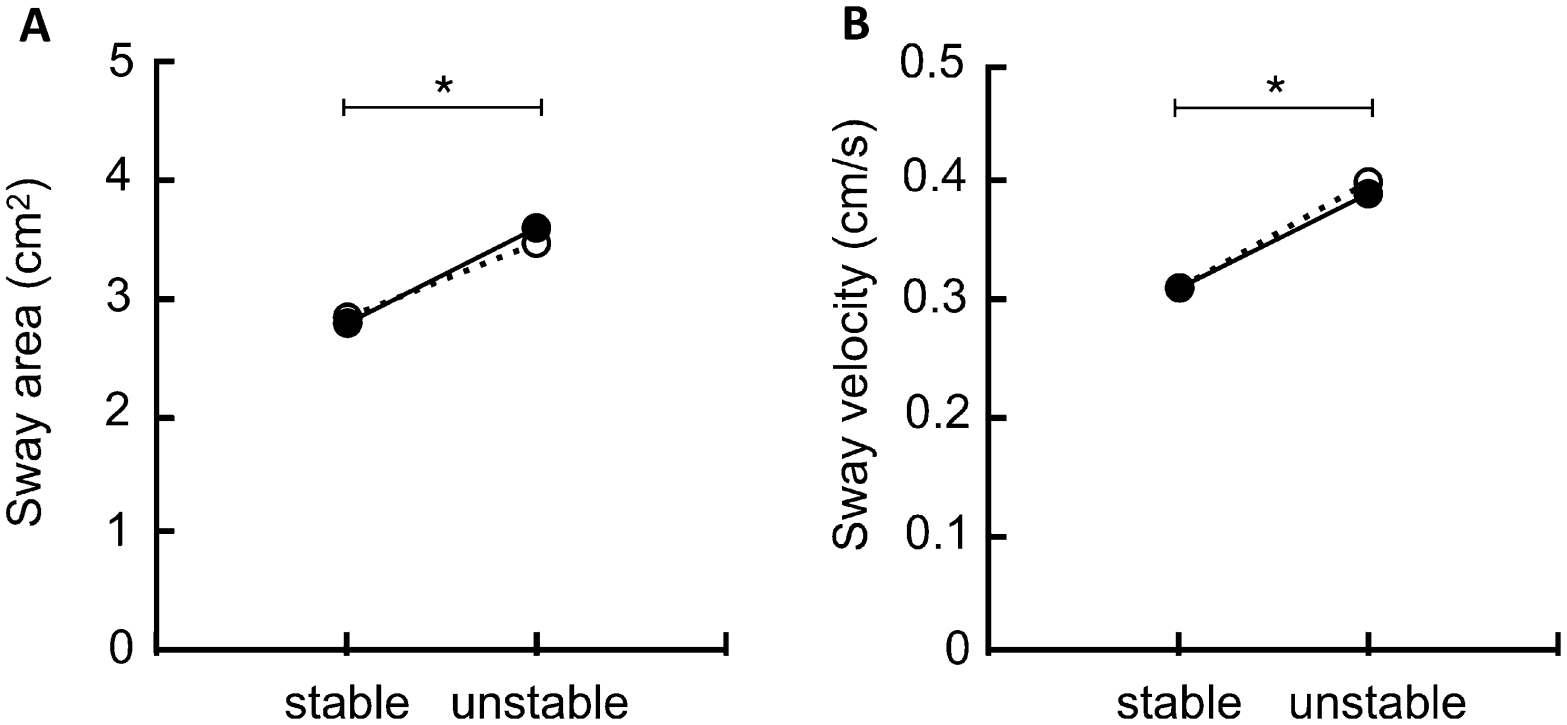
Mean values for the area of sway (in cm^2^) and sway velocity (in cm/s) for single-leg stances on the left (SSL, solid line) and right leg (SSR, dashed line) on the stable and unstable surface. Values are averaged over all included repetitions per condition. *Indicates a significant main effect for stability (*p* < .05)

The results of the two-way repeated-measures ANOVA yielded a significant main effect for stability on SV (*F* = 120.372; *p* < 0.001; *η*_*p*_^2^ = 0.858) and AOS (*F* = 76.362; *p* < 0.001; *η*_*p*_^2^ = 0.792), indicating lower values during stable compared to the unstable conditions. However, postural control outcomes did not differ in the repeated design comparing left and right leg (Fig. 1).

### Cortical activity

**Fig. 2:**
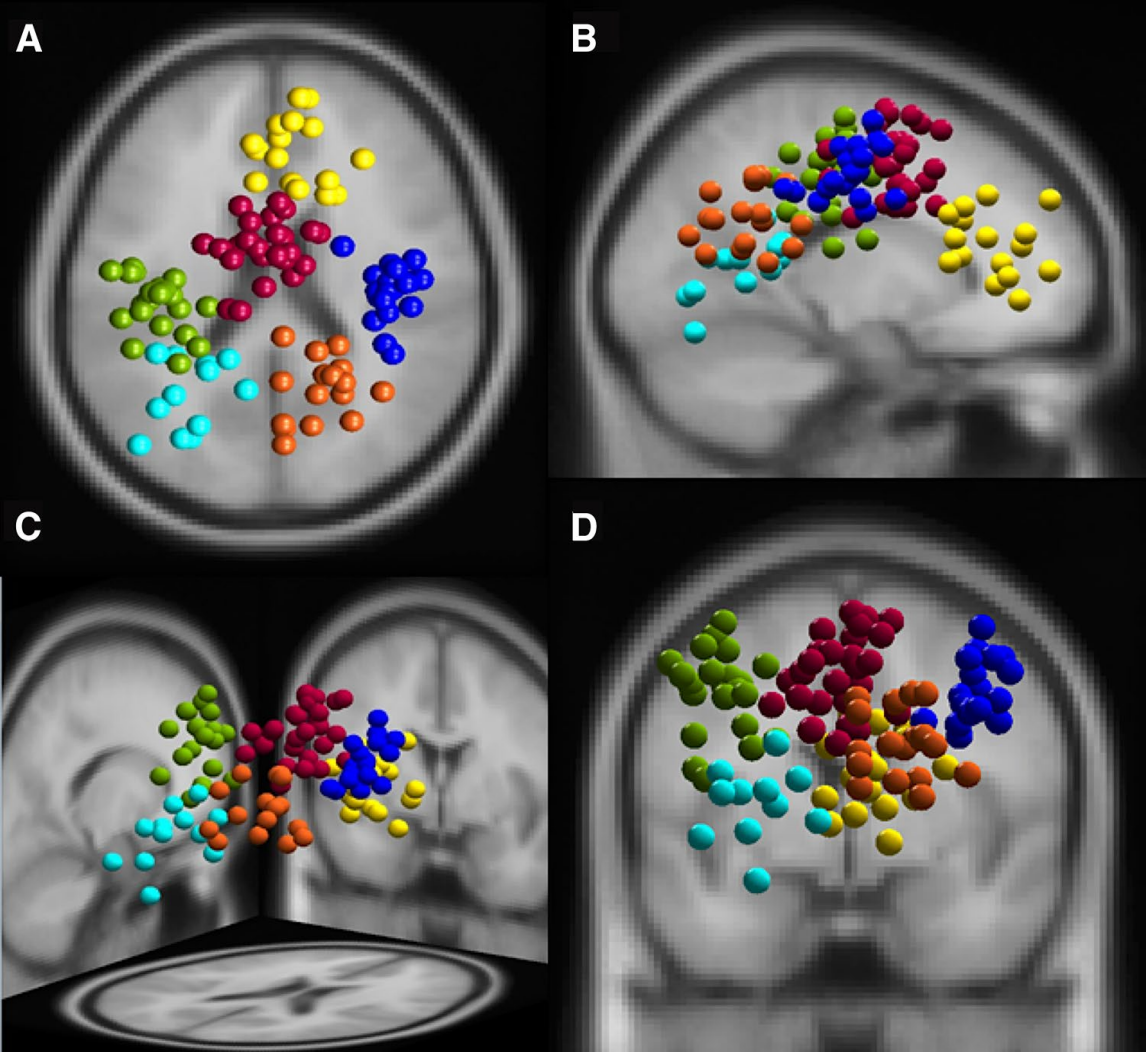
Overview of brain clusters revealed from the independent component analysis. Yellow, prefrontal (16 ICs, RV = 5.86%); red, fronto-central (27 ICs, RV = 5.57%); green, left motor (22 ICs, RV = 4.97%); navy, right motor (19 ICs, RV = 4.20%); cyan, left parieto-occipital (12 ICs, RV = 4.83%); orange, right parieto-occipital cluster (17 ICs, RV = 3.26%).

K-means clustering revealed six clusters of functional brain components, with at least 50% of the sample contributing at least one IC to each cluster. Figure 2 presents the estimated dipoles of brain components assigned to prefrontal (12 participants, 16 ICs), fronto-central (16 participants, 27 ICs), left-motor (16 participants, 22 ICS), right-motor (16 participants, 19 ICs), left-parieto-occipital (10 participants, 12 ICs) and right-parieto-occipital cluster (14 participants, 17 ICs).

#### Theta power

The results of the two-way repeated-measures ANOVA revealed a significant main effect for stability in the fronto-central cluster (*F* = 18.016; *p* < 0.001, *η*_*p*_^2^ = 0.38), where theta power increased from the stable to the unstable condition. An overview of power spectral density at all four experimental conditions is given in Table 1.

**Table 1:** Mean power spectral density with standard deviation (SD) for theta, alpha-1 and alpha-2 frequency bands, given as power [10 × log10 (µV^2^/Hz)]

Cluster	Condition	Theta mean and SD	LEG	STAB	INT	Alpha-1 mean and SD	LEG	STAB	INT	Alpha-2 mean and SD	LEG	STAB	INT
Prefrontal	SSR	27.03 ± 3.17	.79	0.59	.36	24.82 ± 3.47	.44	.25	.39	22.40 ± 2.93	.34	.16	.53
	SSL	27.08 ± 3.17				24.83 ± 3.29				22.38 ± 2.83			
	SUR	27.13 ± 3.23				25.02 ± 3.55				22.53 ± 2.93			
	SUL	27.04 ± 3.23				24.88 ± 3.37				22.42 ± 2.94			
Fronto-central	SSR	26.78 ± 4.73	.72	< .01*	.37	24.38 ± 4.43	.80	.96	.39	23.08 ± 4.34	.98	.14	.30
	SSL	26.69 ± 4.62				24.45 ± 4.47				23.03 ± 4.21			
	SUR	27.19 ± 4.93				24.43 ± 4.41				22.89 ± 4.24			
	SUL	27.21 ± 4.97				24.39 ± 4.33				22.95 ± 4.23			
Left-motor	SSR	25.07 ± 2.71	.98	.97	.41	25.40 ± 3.90	.74	.02*	.28	26.80 ± 4.57	.18	.11	.04*
	SSL	25.14 ± 2.63				25.35 ± 3.78				26.22 ± 4.29			
	SUR	25.14 ± 2.83				24.91 ± 3.73				26.20 ± 4.47			
	SUL	25.07 ± 2.65				25.11 ± 3.46				26.26 ± 4.16			
Right-motor	SSR	24.90 ± 2.70	.67	.83	.72	25.18 ± 4.33	.67	< .01*	.17	26.74 ± 5.14	.67	.16	< .01*
	SSL	24.84 ± 2.55				25.26 ± 4.19				27.38 ± 5.11			
	SUR	24.87 ± 2.61				24.88 ± 3.95				26.89 ± 4.96			
	SUL	24.84 ± 2.57				24.64 ± 3.86				26.45 ± 4.81			
Left-parieto-occipital	SSR	26.31 ± 3.74	.45	.43	.38	27.92 ± 6.09	.58	.07	.62	28.13 ± 5.92	.99	.72	.88
	SSL	26.31 ± 3.31				27.71 ± 5.26				28.11 ± 5.57			
	SUR	26.32 ± 3.53				27.36 ± 5.65				28.04 ± 5.88			
	SUL	26.12 ± 3.33				27.29 ± 5.15				28.06 ± 5.73			
Right-parieto-occipital	SSR	25.84 ± 3.67	.70	.88	.11	26.80 ± 5.25	.90	< .01*	.25	26.60 ± 5.17	.72	< .01*	.64
	SSL	25.87 ± 3.45				26.90 ± 5.00				26.69 ± 5.16			
	SUR	25.92 ± 3.53				26.30 ± 4.68				26.29 ± 4.98			
	SUL	25.82 ± 3.42				26.16 ± 4.50				26.29 ± 4.80			

*Significant result (*p* < .05) for comparisons of left/right standing leg (LEG) or surface stability (STAB), as well as the overall interaction effect (INT)

#### Alpha-1 power

Further, ANOVA revealed a main effect for the stability of alpha-1 power in the left-motor (*F* = 5.67; *p* = 0.02; *η*_*p*_^2^ = 0.16), the right-motor (*F* = 8.304; *p* = 0.008; *η*_*p*_^2^ = 0.27) and the right-parieto-occipital (*F* = 9.613; *p* = 0.004; *η*_*p*_^2^ = 0.23) clusters, as power decreased from stable to unstable conditions.

#### Alpha-2 power

ANOVA indicated an interaction effect for stability and leg in the left-motor cluster (*F* = 4.412; *p* = 0.045, *η*_*p*_^2^ = 0.13). Post-hoc paired-samples *t* tests revealed that alpha-2 power was higher during stable compared to unstable single-leg stance on the right leg (*p* = 0.006, *t* = 2.98, d = 0.131) but a of power values during stable compared to the unstable condition on the left leg did not reveal any difference (*p* = 0.598). Further, post-hoc tests demonstrated higher values for alpha-2 during single-leg stance on the stable surface on the right compared to the left leg (*p* = 0.044, *t* = 2.111, d = 0.128) in the left motor cluster, but not between unstable stance on the right compared to the left leg *p* = 0.775). In the right-motor cluster, ANOVA also yielded a significant interaction effect for stability and leg (*F* = 16.156; *p* = 0.001, *η*_*p*_^2^ = 0.41). Post-hoc paired samples t tests revealed that alpha-2 power was higher during stable compared to unstable SLS on the left leg (*p* = 0.005, *t* = 3.128, d = 0.183) but did not differ during stable compared to the unstable condition on the right leg (*p* = 0.598). Additionally, post-hoc tests revealed a trend towards higher values for alpha-2 during single-leg stance on a stable surface on the left compared to the right leg (*p* = 0.051, *t* = 2.057, d = 0.126) in the left motor cluster, while during unstable conditions revealed a trend towards higher values on the right compared to the left leg (*p* = 0.062, *t* = 1.959, d = 0.089) was observed.

## Discussion

The present study aimed to explore leg-dependent patterns of cortical activation during single-leg stance in both stable and unstable conditions. Task difficulty appeared to increase from stable to unstable surface, as indicated by increasing postural sway and sway velocity. Furthermore, EEG data revealed significantly increased fronto-central theta band power in unstable conditions, while unstable conditions yielded reduced spectral power in the alpha-1 and alpha-2 frequency band in motor and parietal clusters. With regards to leg-dependent cortical contributions to postural control during single-leg stance, a mirrored interaction effect of stance leg and stability in contralateral brain clusters was observed.

### Theta power

The current findings revealed that theta power was significantly increased for the fronto-central cluster when participants were standing on an unstable surface. Theta band oscillations are typically prominent in the ventral frontal cortex of the brain and may represent attentional processing demands (Sauseng et al. 2005). Recent findings suggest that theta oscillations may not only be functionally assigned to ventral regions of the frontal cortex, but also the dorsal regions (Töllner et al. 2017). Töllner and colleagues (2017) linked theta synchronization within a midline frontal cluster to conflict-induced information processing. In line with these findings, Hülsdünker et al. (2015) associated fronto-central theta synchronization (FCz electrode) with increased error detection in consequence of elevated postural demands. In the present investigation, the observed increased body sway demonstrated that the postural equilibrium was perturbed by the unstable base of support, leading to increased error detection and processing. Therefore, it maybe speculated that the increase in fronto-midline theta power along with increased postural sway may represent higher attentional demands and error processing to control for postural equilibrium (Fournier et al. 1999; Slobounov et al. 2009; Sipp et al. 2013; Gebel et al. 2020).

### Alpha power

Analysis of alpha power during the experimental conditions indicated a decrease in alpha-1 and alpha-2 power from stable to unstable conditions. Previous investigations associated these desynchronizations in alpha power with task-specific sensorimotor processing to counteract postural instability (Del Percio et al. 2009; Hülsdünker et al. 2015; Gebel et al. 2020). Accordingly, the present study demonstrated that spectral power in the alpha-1 and alpha-2 frequency band significantly decreased in bilateral motor and right parieto-occipital clusters with increased postural instability. It has been stated that greater amplitudes of alpha oscillations may be related to the active inhibition of non-essential neuronal processing (Pfurtscheller and Lopes 1999). Consequently, centroparietal alpha decrease has been associated with a task-specific release of cortical inhibition as well as increased excitability of neuronal assemblies with regards to sensory processing (Gevins et al. 1997; Pfurtscheller and Lopes 1999; Del Percio et al. 2007; Slobounov et al. 2008; Babiloni et al. 2014). Several investigations have already reported reductions in alpha-1 and alpha-2 power with increasing task difficulty across centro-parietal areas (Slobounov et al. 2009; Hülsdünker et al. 2015; Gebel et al. 2020). As standing on an unstable surface increases the degrees of freedom in postural stability, more compensatory motion can be observed by means of body sway. Consequently, sensorimotor brain areas are required to actively process task-specific postural information, which may be expressed by the reduced alpha-2 activity.

**Fig. 3:**
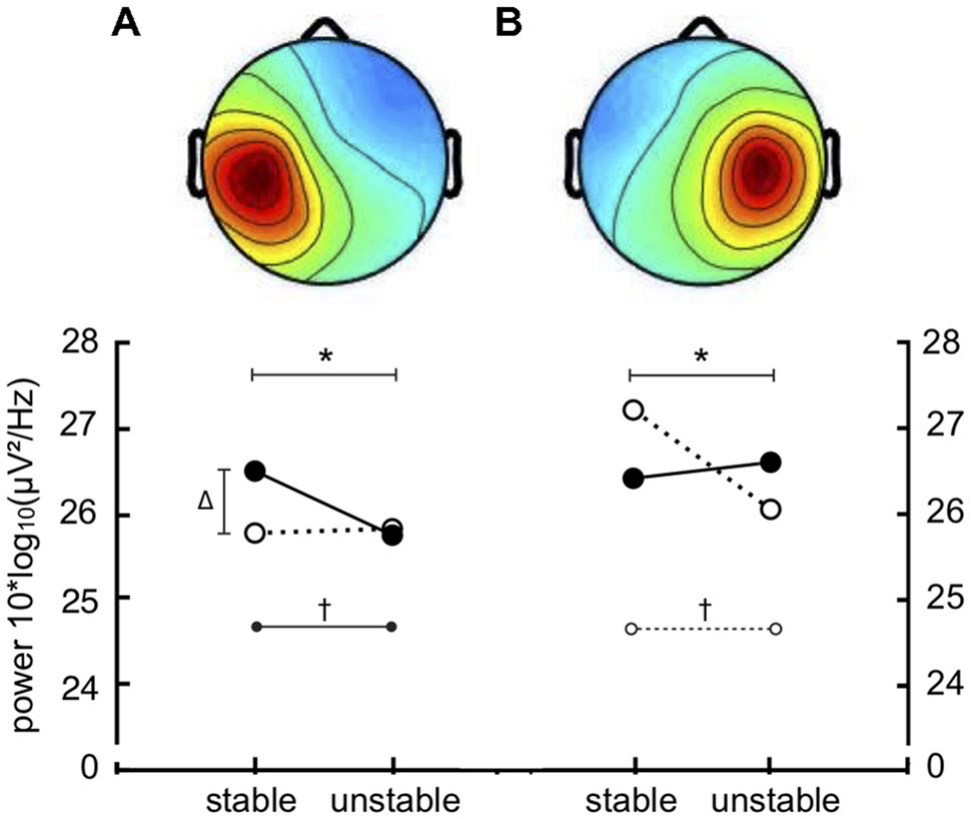
Lateralization effect within the alpha-2 band (10–12 Hz) in the left (**a**) and right (**b**) motor clusters comparing the four different conditions of single-leg stance on stable surface left (SSL) and right (SSR) as well as on unstable surface left (SUL) and right (SUR). Data is given as power [10 × log10 (µV^2^/Hz)]. Solid lines indicate stance on the right leg, dashed lines on the left leg. * = significant interaction effect (*p* < .05) between stance leg and surface stability, Δ = #, significant difference (*p* < .05) between single-leg stance stable left and unstable left. ß, significant difference (*p* < .05) between single-leg stance stable left and stable right

While previous studies have typically not considered leg-depended cortical activity related to postural control, the novel approach of the present study was to examine hemispheric differences in cortical activation in relation to the stance leg. Interestingly, EEG power analysis demonstrated an interaction pattern for stance leg and surface stability in bilateral brain motor areas, while modulations of cortical activity in parietal and frontal brain areas did not demonstrate specificity towards stance leg. While alpha-2 power did not differ in ipsilateral motor areas between stable and unstable conditions, alpha-2 decreased in both motor clusters contralateral to the stance leg from stable to unstable surface (Fig. 3). Moreover, as this interaction effect was particularly pronounced in the contralateral hemisphere, lateralized contributions of contralateral motor areas to the standing leg may be required to control for postural stability. As proprioception and motor control of lower limbs is majorly represented in contralateral motor areas (Kapreli et al. 2006), altered activation of this area may demonstrate changes of the sensorimotor demands to the stance leg in varying postural tasks. In line with this, Cimadoro and colleagues (2013) reported greater activation of stance leg muscles during more challenging balance conditions as derived from electromyography. Thus, a gradual increase in task complexity can be assumed, indexed by a higher degree of contralateral motor cortex activation and stance leg muscle activation. As previous reports of cortical contributions to single-leg stance majorly derived their findings from midline electrodes (Slobounov et al. 2009; Hülsdünker et al. 2015), the present findings provide a deeper insight into the cortical mechanism underlying postural control. While the ipsilateral motor cortex did not show significant modulations with surface stability, it might be suggested that the demands on the non-weight-bearing leg may not change when postural demands increase. As Baumeister et al. (2008) reported that active control of target knee angles induces changes in alpha frequency power, future studies may also examine how different instructions on the position of the non-weight-bearing leg influences ipsilateral cortical activity.

### Limitations

Some methodological limitations may be considered when interpreting the present findings. A methodological issue inherently related to EEG assessments is the limited spatial resolution (Mehta et al. 2014). Although ICA was applied to reduce volume conduction effects, IC dipoles solely display an approximation of the real cortical source of the signal and exact spatial assignment of EEG signals should be considered with caution (Jungnickel and Gramann 2016). Another limitation of the present study may be related to the physical characteristics of the sample. It has been shown that athletes from different sports show less postural sway (Paillard et al. 2006; Kiers et al. 2013) in consequence of a well-established perception–action coupling by long-term training (Gautier et al. 2008). Since the current approach did not control for sporting participation and experience, the heterogeneous physical and motor background may have influenced postural stability and consequently cortical processing.

Furthermore, exploratory EEG approaches in a mobile paradigm exhibit methodological limitations which may affect source space analysis. In the presence of multiple non-brain sources, a mixture of real brain and non-brain signals may be represented in several ICs. On the one hand, functional brain components may therefore not be detectable for every single participant and could limit the analysis through a mismatch of physiologically plausible ICs within the model (Artoni et al. 2014). On the other hand, participants may contribute more than a single functional IC to the clusters, which might be related to time-dependent characteristics within spatially closely tied cortical areas. As similarly reported in previous studies (Peterson and Ferris 2018; Solis-Escalante et al. 2019), the clusters of the present analysis did not demonstrate an equal distribution of the entire study sample. As EEG source modeling is essentially based on computational derivations of the analyzed real signal and does not detect specific, a-priori defined ICs, single subjects did not contribute to one of the clusters and some clusters partially contained multiple ICs per participant. Future studies may develop optimized approaches of source space analysis in mobile EEG experiments to increase the statistical validity of the observed cortical phenomena.

The main finding of the present study was an increase in contralateral brain motor areas when standing on one leg on an unstable surface. As previous studies suggested a potential right-sided scalp preponderance of cortical processing during postural control tasks (Dimitrov and Gavrilenko 1996), future studies should further examine the effect of the suggested preponderance on leg-dependent modulations of cortical activity.

## Conclusion

In summary, the present study revealed leg-dependent patterns of cortical activity with increasing postural instability during single-leg stance in healthy subjects. In line with previous studies, surface instability and concurrently increasing postural sway may entail higher attentional demands or error processing to control for postural equilibrium. Beyond that, the postural instability and task difficulty may also be associated with leg-dependent patterns of cortical activity in the contralateral hemisphere to the standing leg, indicating enhanced contributions of the related motor areas to maintain postural stability in unstable surface conditions. These findings may help to explore postural deficiencies in patients with various impairments and to develop neurophysiological assessments for their functional recovery. However, further investigations are required to determine the functional significance of leg-dependent cortical modulations related to postural control.

## Data Availability

The datasets generated during and/or analysed during the current study are available from the corresponding author on reasonable request.
